# Analysis of KRAS Mutation Subtype in Tissue DNA and Cell-Free DNA Using Droplet Digital PCR and the Function of Cell-Free DNA as a Recurrence Predictive Marker in Pancreatic Cancer

**DOI:** 10.3390/biomedicines9111599

**Published:** 2021-11-02

**Authors:** Eunsung Jun, Bonhan Koo, Eo Jin Kim, Dae Wook Hwang, Jae Hoon Lee, Ki Byung Song, Woohyung Lee, Yejong Park, Sarang Hong, Yong Shin, Song Cheol Kim

**Affiliations:** 1 Department of Convergence Medicine, Asan Institute for Life Sciences, University of Ulsan College of Medicine and Asan Medical Center, Seoul 05505, Korea; kokoejko@gmail.com; 2 Division of Hepatobiliary and Pancreatic Surgery, Department of Surgery, Asan Medical Center, AMIST, University of Ulsan College of Medicine, Seoul 05505, Korea; dwhwang@amc.seoul.kr (D.W.H.); hbpsurgeon@gmail.com (J.H.L.); mtsong21c@naver.com (K.B.S.); ywhnet@gmail.com (W.L.); blackpig856@gmail.com (Y.P.); 8thofnovember@hanmail.net (S.H.); 3 Department of Biotechnology, College of Life Science and Biotechnology, Yonsei University, Seoul 03722, Korea; qhsgksdlek@naver.com

**Keywords:** pancreatic cancer, cell-free DNA, KRAS mutation, G12D, G12V, mutation burden, recurrence-free survival, predictive marker

## Abstract

KRAS mutation is a major regulator in the tumor progression of pancreatic cancer. Here, we compared the frequency and mutation burden of KRAS mutation subtypes with paired tumor tissue and blood in patients and examined their clinical significance. DNA from tumor tissues and cell-free DNA (cfDNA) from preoperative blood were obtained from 70 patients with pancreatic cancer. Subtypes and mutation burdens of KRAS G12D and G12V mutations were evaluated using droplet digital PCR. Comparing the presence of mutations in tissue, accumulative and simultaneous mutations of G12D or G12V were identified of 67 (95.7%), and 48 patients (68.6%). Conversely, in blood, they were only identified in 18 (25.7%) and four (5.7%) patients; respectively. Next, comparing the mutation burden in tissue, the mutation burden varied from less than 0.1 to more than five, whereas that of cfDNA in blood was mostly between one and five, as cases with a mutation burden lower than 0.1 and higher than five were rare. Finally, the presence of the G12V mutation alone in cfDNA and the combination of the G12V mutation with elevated CA 19-9 levels were associated with poor recurrence-free survival. These fundamental data on the KRAS mutation subtypes and their clinical significance could support their potential as predictive markers for postoperative recurrence.

## 1. Introduction

Pancreatic cancer is a refractory carcinoma with poor prognosis. Its 5-year survival rate is only 8–10%, and the incidence continues to increase; thus, pancreatic cancer is predicted to be a carcinoma with very high mortality rate even after 2030 [1,2,3]. Recently, preoperative chemotherapy has been reported to increase the recurrence-free survival (RFS) and overall survival (OS) after surgery; however, the effectiveness of neoadjuvant chemotherapy is limited and it remains difficult to establish an optimized therapeutic plan compared to other carcinomas [4,5,6].

For a therapeutic approach to pancreatic cancer patients, it is necessary to understand and study the genetic mutations that cause tumor development and induce progression. During the transformation of normal pancreatic cells into cancer cells, mutations such as those in RAS, CDKN2A, TP53, and SMAD4 occur, among which the RAS mutation is one of the earliest [7,8]. Among mutationally activated RAS genes (HRAS, KRAS, and NRAS), KRAS is the predominant isoform and is exclusively mutated in pancreatic ductal adenocarcinoma (PDAC) [8]. KRAS mutations in normal cells result in a protein that is locked in a continuously active state, unable to hydrolyze GTP and thus promoting persistent signaling to downstream effectors [9,10]. Mutant KRAS expression under acinar and ductal promoters results in ductal lesions reminiscent of pancreatic intraepithelial neoplasia (PanIN) and in mixed acinar and ductal carcinomas [11]. In particular, KRAS activation in pancreatic cancer, also acting as a master regulator, has been reported to promote cancer progression by influencing the properties of several tumor microenvironment components and by recruiting fibroblasts, macrophages, neutrophils, and lymphocytes, among other cells [12]. Thus, KRAS mutation is the main hurdle in pancreatic cancer treatment and is a major target for the development of therapeutic agents, as it affects tumor development, rapid progression, and drug resistance [8,13,14].

KRAS mutation is not limited to pancreatic cancer, and although it is frequently expressed in other malignancies, such as non-small-cell lung carcinoma and colorectal cancer, there are differences in the location of the mutation. In pancreatic cancer, unlike in other tumors, mutations have been reported mostly in codon 12, and most of them are G12D and G12V mutations [15,16]. The survival rate of patients with pancreatic cancer with KRAS mutation is worse than that of patients with pancreatic cancer with wild-type KRAS, and is reported to be worse in cases with the G12D mutation than in those with the G12V mutation. The KRAS G12D mutation has been reported as a prognostic factor in patients with advanced pancreatic adenocarcinoma [17,18,19]. The difference between these KRAS subtypes can be attributed to the difference in the structure of the amino acid binding to GTP; the original glycine residue is changed to aspartic acid or valine, which alters signal activation, such as that related to Raf, ERK, and MEK, as well as changes in the tumor matrix, resulting in changes in response to treatment [16,20,21,22]. Therefore, the KRAS mutation subtype is an important factor as it can determine and predict the patient’s response to pancreatic cancer surgery and chemotherapy. However, the correlation between mutation subtype and clinical status remains unclear in terms of the number of enrolled patients and disease status (resected vs. locally advanced vs. metastasis), the tissue source (surgical specimen vs. endoscopic biopsy), and the method of mutation detection (sequencing vs. real-time polymerase chain reaction vs. droplet digital polymerase chain reaction (ddPCR) vs. next-generation sequencing) [23,24].

In this study, we tried to determine the KRAS mutation subtype and the extent of the mutation burden in cell-free DNA (cfDNA) as well as in the tumor tissues of patients with pancreatic cancer using ddPCR. We also examined whether these quantitative data were related to clinicopathological findings and whether their combination with CA 19-9, a pancreatic cancer marker could help to predict patient prognosis.

## 2. Materials and Methods

### 2.1. Study Design

This retrospective study complied with the Declaration of Helsinki and was reviewed and approved by the Institutional Review Board (IRB) of Asan Medical Center (IRB No. 2019-0631, approval date (17 May 2019)). Patients who underwent surgery for pancreatic cancer in the hepato-biliary and pancreatic surgery division of Asan Medical Center were enrolled in this study, and informed consent was obtained from them. We used paired tumor tissues and preoperative blood samples from 70 patients who were diagnosed with PDAC. The bio-specimens and data used in this study were provided by the Asan Bio-Resource Center, Korea Biobank Network (BRC No. 2019-8(187)).

### 2.2. Clinical Information

Each patient was followed up for at least 4 years, and the medical records of patients were retrospectively reviewed for clinicopathological characteristics. Age, sex, preoperative laboratory findings, pathological findings, history of neoadjuvant chemotherapy, and tumor recurrence during follow-up were recorded. Preoperative blood tests included measurements of white blood cell (WBC), neutrophil, monocyte, and platelet counts and hemoglobin (Hb), aspartate aminotransferase (AST), alanine aminotransferase (ALT), total protein, albumin, blood urea nitrogen (BUN), creatinine, carbohydrate antigen 19–9 (CA 19-9), and carcinoembryonic antigen (CEA) levels. The surgical procedure (pancreaticoduodenectomy (PD) or distal pancreatectomy and splenectomy (DPS)) was chosen according to the tumor location and extension. Pathological findings included tumor size, tumor differentiation, lymphovascular invasion, perineural invasion, and metastatic lymph nodes. Tumor, node, and metastasis (TNM) staging was conducted in accordance with the eighth edition of the American Joint Committee on Cancer manual [25].

Furthermore, during postoperative surveillance, computed tomography (CT) and CA 19-9 levels were checked every 3 months during the first 2 postoperative years and every 4–6 months thereafter.

### 2.3. DNA Preparation and ddPCR Assay

Tissues stored in cryotubes were cut into pieces weighing <30 mg, and DNA was extracted from lysed tissue samples using a DNeasy Mini Kit (ID: 69504, Qiagen, Germantown, MD, USA) according to the manufacturer’s instructions. Furthermore, the cfDNA in plasma was extracted using the microfluidic cfDNA sampling platform as previous described [26]. The microfluidic platform (85 mm × 70 mm × 5 mm) is manufactured by assembling a thin film, which forms the top and bottom of the platform, and a double-sided tape, which forms the channel designed using AutoCAD (Autodesk, Inc., San Rafael, CA, USA). The inner surface of the microfluidic channel was treated with oxygen plasma (O_2_: 80 sccm; power: 100 W; time: 10 min) and then immersed in a solution of 2% 3-aminopropyl (diethoxy) methylsilane in distilled water for 60 min at 65 °C to modify the amine group. Next, 1.3 mL of a mixture containing 1 mL of plasma and 300 µL of dimethyl dithiobispropionimidate (DTBP, 100 mg/mL) was injected into the modified platform at 100 µL/min and incubated for 20 min. The cfDNA was captured via covalent binding and electrostatic coupling with DTBP and the amine groups on the inner surface of the platform. The captured cfNA and circulating tumor DNA were then extracted using a high-pH (pH 10.4) elution buffer. The concentration and purity of DNA were confirmed using a NanoDrop-2000 Spectrophotometer (ND-2000, Thermo Fisher Scientific, Waltham, MA, USA), and the DNA was stored at −80 °C until needed for mutation analysis. Extracted DNA was tested using ddPCR (QX200 Droplet Digital PCR System; Bio-Rad Laboratories Inc., Hercules, CA, USA) using the Prime PCR KRAS mutant assays (Bio-Rad, dHsaCP2000001 (G12D), dHsaCP2000005 (G12V), and corresponding WT assays (dHsaCP2000002 (G12D), dHsaCP2000006 (G12V)). The reaction mixtures (final volume, 20 µL) comprised extracted DNA (2 µL), 2× SuperMix for probe (10 µL), KRAS mutant assays probe (1 µL), KRAS WT assays probe (1 µL), and distilled water (6 µL). The mixture was loaded into a disposable droplet generator cartridge (Bio-Rad), and 70 µL of droplet generation oil for the primer (Bio-Rad) was loaded into each of the eight oil wells. The cartridge was then placed inside the QX200 droplet generator (Bio-Rad), which partitioned each tissue sample into ~22,000 droplets per tissue sample. When droplet generation was completed, the droplets were transferred to a 96-well PCR plate. The plate was heat-sealed with foil and placed in a conventional thermal cycler (Veriti™ 96-Well Thermal Cycler; Thermo Fisher Scientific, Waltham, MA, USA) using the following reaction conditions: 95 °C for 10 min (1 cycle); 94 °C for 30 s and 55 °C for 1 min (40 cycles); 98 °C for 10 min (1 cycle); and 4 °C hold. The thermally cycled droplets were then read individually using a QX200 droplet-reader (Bio-Rad, Hercules, CA, USA). Samples were transferred to the QX200 for the measuring of the fluorescence of the mutant probe labeled with 6-fluorescein amidite and the wild-type probe labeled with hexachloro-fluorescein. Quanta Soft software (version 1.7; Bio-Rad, Hercules, CA, USA) was used to analyze the raw fluorescence amplitude and to obtain the fractional abundance for KRAS mutations [23]. The criterion set for mutation positivity was 0.01% or more of fractional abundance [27,28,29].

### 2.4. Statistical Analysis

Data are expressed as the mean ± standard error for continuous variables and as frequency for categorical variables. Student’s *t*-test, the chi-squared test, or the Kruskal–Wallis test were used to analyze the differences between the values of continuous and categorical variables. The OS and RFS were determined using the Kaplan–Meier method with the log-rank test. All statistical analyses were performed using the Statistical Package for the Social Sciences (SPSS) version 21.0 (IBM Corp., Armonk, NY, USA).

## 3. Results

### 3.1. Clinicopathological Features of Enrolled Patients

In total, 70 patients who underwent surgery for pancreatic cancer were included in the study to determine the presence of KRAS mutations in tissues and blood. In the preoperative blood test, the average ALT level increased to 47.6 (IU/L) and the average CA 19-9 expression increased to 674.5 IU/mL; however, except for these, there were no other specific laboratory findings. Only nine patients received neoadjuvant chemotherapy before surgery, and 48 (68.6%) and 22 (31.4%) patients received PD and DPS surgery, respectively, depending on the tumor location. According to the results of histopathological examination, the average tumor size was 3.7 cm, and the T2 stage and N1 stage were the most dominant (Table 1).

### 3.2. Presence of KRAS Mutation in Paired Tissue DNA of Tumor and cfDNA in Blood

**Figure 1:**
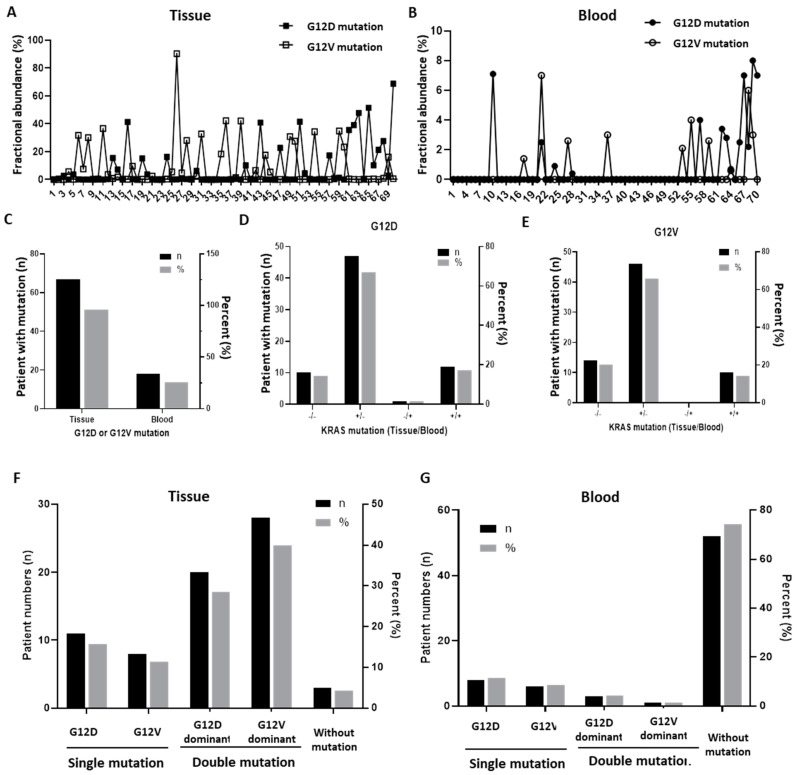
The presence of KRAS G12D/G12V mutations in tissues and blood (*n* = 70). (**A**) Fractional abundance of KRAS G12D and G12V mutations identified in tumor tissues. (**B**) Fractional abundance of KRAS G12D and G12V mutations identified in blood. (**C**) Overall frequency of the expression of G12D or G12V mutations in tissues and blood. (**D**) Frequency of the expression of the G12D mutation by resource in tissues and blood. (**E**) Frequency of the expression of the G12V mutation by resource in tissues and blood. (**F**) Comparison of the dominance of the expression of G12D and G12V mutations in tissues. (**G**) Comparison of the dominance of the expression of G12D and G12V mutations in blood.

First of all, the fractional abundance of KRAS G12D and G12V mutations in paired tumor tissues and blood was analyzed; the individual mutation values are described in Table S1. In addition, the fractional abundance of mutations in tissue is shown in Figure 1A, and the results of cfDNA analysis in blood are shown in Figure 1B. Upon comparing the overall trends through tumor and blood groups, mutations were confirmed in 67 cases (95.7%) except for three cases in tissue, whereas mutations were confirmed in only 19 cases (27.1%) in blood. Furthermore, the fractional abundance of mutation was more than three times higher in tissue (95.7%) than in blood (25.7%) (Figure 1C).

According to the KRAS mutation subtype (G12D and G12V), the presence or absence of mutations in the tissues and blood of each patient was analyzed, respectively (Figure 1D,E). The tissue and blood groups showed a similar trend regardless of the mutation subtype, which was most often detected only in tissue, followed by simultaneous detection in tissue and blood. Moreover, detection in both tissue and blood was negative in approximately 10% of the cases, and detection only in blood was even rarer.

Next, according to the DNA resource (tissue and blood), the presence, dominance, or absence of G12D or G12V mutations in each patient was analyzed, respectively (Figure 1F,G). In the tissues, 11 patients (15.7%) had only the G12D mutation, eight patients (11.4%) had only the G12V mutation, and 48 patients (68.6%) had both mutations. On the other hand, in blood, eight patients (11.4%) only had the G12D mutation, six patients (8.6%) had only the G12V mutation, and four patients (5.7%) had both mutations.

### 3.3. Correlation of KRAS Mutation Burden with Clinicopathological Status

**Figure 2:**
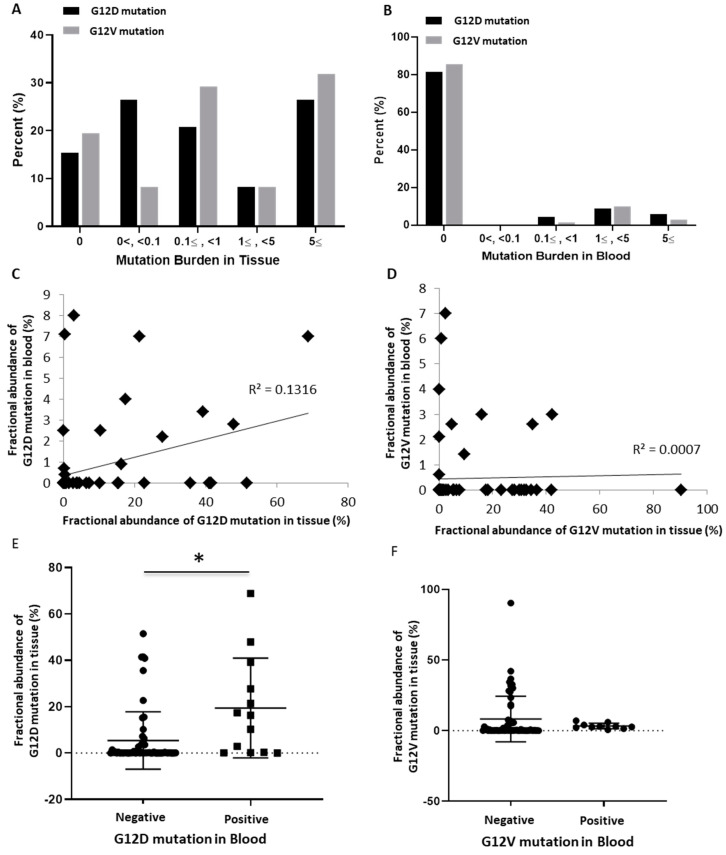
The abundance of KRAS G12D/G12V mutations in tissues and blood (*n* = 70)**.** (**A**) Comparison between G12D and G12V mutations according to the degree of fractional abundance in tissues. (**B**) Comparison between G12D and G12V mutations according to the degree of fractional abundance in blood. (**C**) Correlation of the G12D mutation burden in tissues and blood (R^2^ = 0.1316). (**D**) Correlation of the G12V mutation burden in tissues and blood (R^2^ = 0.0007). (**E**) Mutation burden of tissues according to the presence or absence of the G12D mutation in blood (*; *p* = 0.0024). (**F**) Mutation burden of tissues according to the presence or absence of the G12V mutation in blood (*p* = 0.3386).

Through the fractional abundance of each mutation, the correlation between mutation burden and clinicopathological status was analyzed. The mutation burden of G12D and G12V mutations in tissue DNA ranged from a small amount of less than 0.1 to a high frequency of five or more, with a slight frequency difference between the two mutation subtypes. By contrast, in the cfDNA of blood, no amount less than 0.1 was detected, and the frequency mostly ranged between one and five (Figure 2A,B). In analyzing the linear correlation of mutation burden of each patient between tissue and blood, the G12D mutation showed a slightly more positive correlation than the G12V mutation (Figure 2C,D). Thus, when the G12D mutation was present in the blood, the fractional abundance of the G12D mutation in the tissue was significantly increased (*p* < 0.05). However, the presence of the G12V mutation in blood did not show a significant correlation with the increase in the tissue G12V mutation burden (Figure 2E,F).

Additionally, the association between each type of mutation burden and various clinicopathological findings was analyzed. There was no significant difference according to the tumor location (head/neck vs. body/tail), T stage, N stage, TNM stage, CA 19-9 value, and the presence of neoadjuvant chemotherapy (Figure S1).

### 3.4. Survival Analysis According to the Site and Subtype of KRAS Mutation

**Figure 3:**
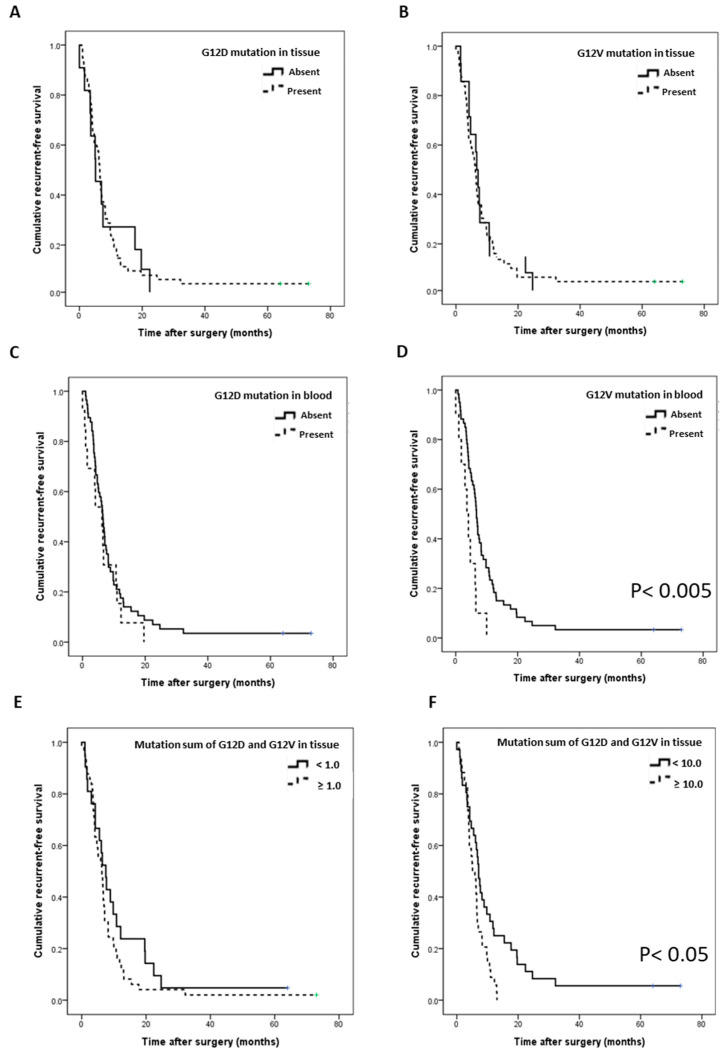
Correlation of the recurrence-free survival (RFS) with the G12D/V mutation (*n* = 70). (**A**) RFS according to the presence of the G12D mutation in tissues. (**B**) RFS according to the presence of the G12V mutation in tissues. (**C**) RFS according to the presence of the G12D mutation in blood. (**D**) RFS according to the presence of the G12V mutation in blood (*p* = 0.004). (**E**) RFS according to the sum of G12D and G12V mutation burden in tissues (more than 1). (**F**) RFS according to the sum of G12D and G12V mutation burdens in tissues (more than 10, *p* = 0.010).

We then analyzed whether there was a difference in the RFS and OS of patients according to the presence and burden of the mutation. In the patients showing G12D or G12V mutations in tissue DNA and in those with G12D mutations in cfDNA, no significant difference was found between RFS and OS (Figure 3A–C, Figure S2A–C). However, when the G12V mutation was detected in cfDNA, RFS and OS were significantly worse (Figure 3D; *p* = 0.004, Figure S2D; *p* = 0.034). Furthermore, analysis of the RFS and OS according to the quantitative difference between G12D and G12V mutation burdens for each tissue showed that the RFS rate was significantly worse in the patient group with the sum of burdens of 10 or higher (Figure 3E,F; *p* = 0.010, Figure S2E,F; *p* = 0.033).

### 3.5. Survival Prediction with the Combination of KRAS Mutation and CA 19-9

**Figure 4:**
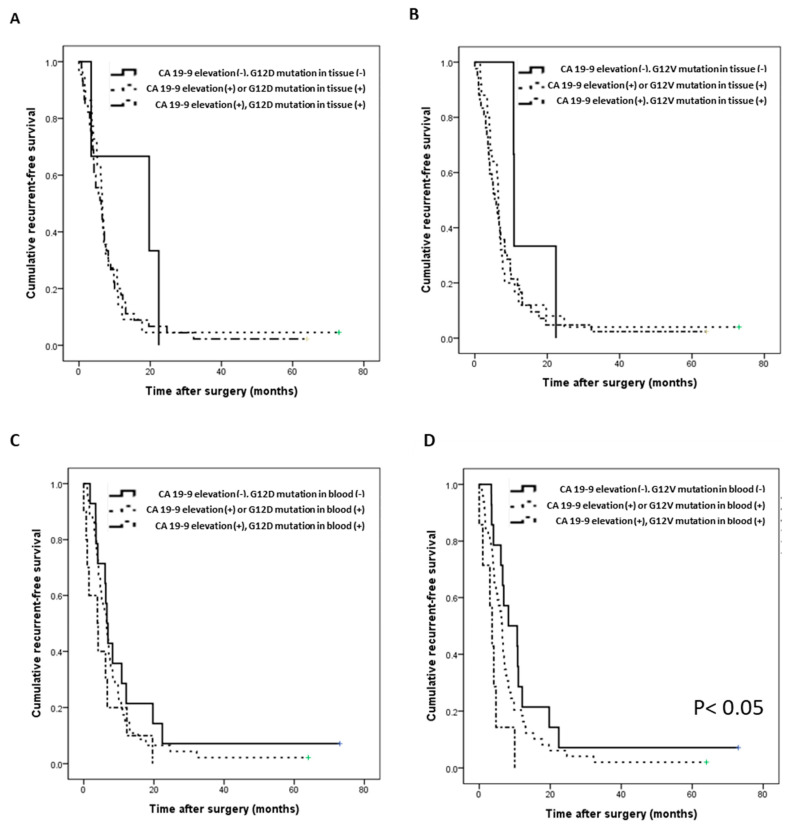
Correlation of the recurrence-free survival (RFS) with the G12D/V mutation and CA 19-9 (*n* = 70). (**A**) RFS according to the combination of the G12D mutation and the CA 19-9 level in tissue. (**B**) RFS according to the combination of the G12V mutation and the CA 19-9 level in tissue. (**C**) RFS according to the combination of the G12D mutation and the CA 19-9 level in blood. (**D**) RFS according to the combination of the G12V mutation and the CA 19-9 level in blood (*p* = 0.014).

We analyzed survival prediction through the combination of KRAS mutation in cfDNA and CA 19-9 expression in plasma as a representative biomarker of pancreatic cancer. First, we confirmed that the OS and RFS of enrolled patients did not differ significantly with an increase in only CA 19-9 expression (Figure S3A,B). Compared with CA 19-9 expression elevation alone or the KRAS mutation alone, the RFS and OS tended to be slightly worse when both were present (Figure 4A–C, Figure S3C–F). However, when the G12V mutation and elevated levels of CA 19-9 were present in blood simultaneously, RFS and OS were significantly decreased (Figure 4D; *p* = 0.014, Figure S3F; *p* = 0.014). Specifically, when the levels of CA 19-9 were low and there was no G12V mutation in blood, the average RFS was 8.2 months; by contrast, when the CA 19-9 levels were high and the G12V mutation in blood was simultaneously present, the average RFS was only 3.6 months (*p* = 0.014, Figure 4D).

## 4. Discussion

Gene mutations are indicators that can be used to predict tumor incidence and progression. It is thus essential to accurately analyze the occurrence, types, and extent of tumor mutations to determine the therapeutic strategy. The technology for analyzing genetic mutations in various human resources has developed very rapidly, and recently many researchers have compared the sensitivities of analysis methods, including PCR, next-generation gene sequencing, and droplet digital PCR, for the analysis of gene mutations. ddPCR is an advanced digital PCR technology with a very high sensitivity that can identify even a mutant with an occurrence of 0.01% [23,30]. When analyzing KRAS mutations in tissues, ddPCR was found to be superior to Sanger sequencing and to the PNA clamping assay in terms of diagnostic sensitivity (100%) and specificity (100%) [23]. Furthermore, upon comparing ddPCR and NGS for KRAS mutation detection in tissue DNA and plasma cfDNA, ddPCR was found to be superior to NGS with respect to the success rate and concordance with the tissue in both analyses [24]. Therefore, we used the ddPCR method, which enables the qualitative and quantitative analysis of the presence and subtypes of KRAS mutations in tumor tissues and in cfDNA in blood.

In this study, we could confirm the presence of G12D or G12V mutations in 95.7% of the tissue DNA and 25.7% of cfDNA from plasma in patients with pancreatic cancer. The detection rate of KRAS in plasma cfDNA was lower than that in tissues, and other studies have reported detection rates in blood of 29.5%, 32–41%, and 68% [30,31,32]. Upon comparing the fractional abundance of mutations between tissue DNA and plasma cfDNA, the mutation burden in tissue DNA showed a very wide range of values, from less than 0.1 to more than five, whereas in the case of cfDNA, almost no mutation burden of less than 0.1 was found. We could assume that when the amount of mutation in the tissue is above a certain standard, or when a large amount of DNA fragments are produced or well secreted into the blood, target DNA can be well detected in plasma. In our results, a weakly positive correlation of the mutation burden was found between tissue and plasma. Furthermore, in the case of the G12D mutation in blood, the mutation burden of G12D in tissue was high (*p* < 0.05); thus, the above assumption could be supported (Figure 2C–E). In particular, the RFS differed depending on the combination of the KRAS G12V mutation and the levels of CA 19-9 in plasma (Figure 4D). Patient prognosis could not be predicted using each variable individually; however, RFS could be predicted through a combination of the two variables. Although CA 19-9 is a marker that is widely used clinically, it can be used more usefully in combination with other biomarkers to evaluate the progression and recurrence of tumors [33,34]. Furthermore, double G12D and G12V mutations were identified, and the higher the sum of these mutations, the lower were the RFS and OS (Figure 3F, Figure S2F). The diversity of these mutations may be the cause of the complex heterogeneity in the tumor microenvironment and may be characteristic of refractory tumors such as pancreatic cancer [35,36,37]. The approach based on the precise analysis of cfDNA is expected to be effective for analyzing the genetic subtype and its related cancer microenvironment and for predicting the progression status and drug response of pancreatic cancer.

In the correlation between KRAS mutations and clinical features, most reports indicate that mutations are associated with a decrease in survival rate [17,27]. In analyses of KRAS mutation subtypes, slightly different results have also been reported, contrary to our findings. For instance, Windon et al. observed that the survival rate in the case of the G12D mutation among KRAS mutations of codon 12 was slightly worse than that in the case of the G12V mutation [17]. Kim et al. reported that the median survival of the G12D mutation group was 12.4 months, whereas the median survival of the G12V group was 16.3 months; although this difference was not statistically significant, the G12D group had a slightly worse survival rate (*p* > 0.05) [38]. However, because these differences can occur depending on tumor progression and treatment, as well as the methods used for DNA extraction, DNA mutation analysis, and statistical analysis, detailed analyses and interpretations are required to obtain reliable conclusions.

This study was a retrospective study targeting patients with pancreatic cancer and has several limitations. Patients were enrolled from a single institute, the number of enrolled patients was rather small (70 patients), few patients were in the advanced stage, and information on adjuvant chemotherapy was not included in the study. Therefore, based on the current research results, it is thought that additional research using tumor resources of various clinical statuses from several more institutions is necessary.

## 5. Conclusions

To predict the progress and treatment response based on the molecular characteristics of the tumor microenvironment, a detailed analysis of the genetic mutations in pancreatic tumors is needed. In this study, we obtained qualitative and quantitative information on the KRAS mutation subtypes in paired tumor tissues and blood and evaluated their function to predict prognosis with CA 19-9 levels. We expect that the results of a precise analysis of mutation subtypes will provide clues to understanding tumor development and the fundamental tumor microenvironment and will facilitate the determination of optimal therapeutic strategies for individual patients.

## Figures and Tables

**Table 1:** Clinical information of the enrolled patients (*n* = 70). (BMI: body mass index; Hb: hemoglobin; WBC: white blood cell; AST: aspartate aminotransferase; ALT: alanine aminotransferase; BUN: blood urea nitrogen; CA 19-9: carbohydrate antigen 19-9; CEA: carcinoembryonic antigen; PD: pancreaticoduodenectomy; DPS: distal pancreatectomy with splenectomy; wel: well; mod: moderate; por: poor; LVI: lymphovascular invasion; PNI: perineural invasion).

Factors		Values
Age (y)	Avg. ± SD (Range)	61.4 ± 10.1 (30–88)
Sex (Female/Male)	N (%)	19/51 (27.1%/72.9%)
BMI(Kg/m^2^)	Avg. ± SD (range)	23.5 ± 3.6 (16.2–35.4)
Neoadjuvant Chemotherapy (Yes/No)	N (%)	61/9 (87.1%/12.9%)
Preoperative Lab		
Hb (g/dL)	Avg. ± SD (range)	12.6 ± 1.8 (8.4–16.5)
WBC (×10^3^ μL)	Avg. ± SD (range)	6.5 ± 2.2 (3.6–16.9)
Neutrophil (%)	Avg. ± SD (range)	59.4 ± 12.7 (27.4–95.4)
Lymphocyte (%)	Avg. ± SD (range)	29.2 ± 10.3 (2.2–51.7)
Monocyte (%)	Avg. ± SD (range)	7.0 ± 2.4 (2.3–13.2)
Platelet (×10^3^ μL)	Avg. ± SD (range)	239.7 ± 95.9 (93.0–624.0)
AST (IU/L)	Avg. ± SD (range)	30.9 ± 23.9 (8.0–161.0)
ALT (IU/L)	Avg. ± SD (range)	47.6 ± 62.2 (7.0–359.0)
Total Protein (g/dL)	Avg. ± SD (range)	6.6 ± 0.7 (3.9–7.9)
Albumin (g/dL)	Avg. ± SD (range)	3.5 ± 0.5 (1.5–4.6)
BUN (mg/dL)	Avg. ± SD (range)	14.0 ± 5.6 (2.0–35.0)
Creatinine (mg/dL)	Avg. ± SD (range)	0.8 ± 0.4 (0.3–3.7)
CA19-9 (U/mL)	Avg. ± SD (range)	674.5 ± 1301.7 (1.1–7480.0)
CEA (ng/mL)	Avg. ± SD (range)	5.8 ± 8.1 (0.6–57.2)
Operation and Pathologic findings		
PD/DPS	N (%)	48/22 (68.6%/31.4%)
Tumor size (cm)	Avg. ± SD (range)	3.7 ± 1.3 (1.6–9.0)
Tumor differentiation (wel/mod/por)	N (%)	3/51/16 (4.3%/72.9%/22.9%)
T stage (T1/T2/T3)	N (%)	2/48/20 (2.9%.68.6%/28.6%)
LVI (absent/present)	N (%)	32/38 (45.7%/54.3%)
PNI (absent/present)	N (%)	7/63 (10.0%/90.0%)
N stage (N0/N1/N2)	N (%)	23/30/17 (32.9%/42.9%/24.3)

## Supplementary Materials

The following are available online at https://www.mdpi.com/article/10.3390/biomedicines9111599/s1, Table S1: The fractional abundance of KRAS G12D and G12V mutations in the tumor tissues and blood (*n* = 70). Figure S1: Correlation of KRAS burden with tumor location, tumor progression, and the presence of neoadjuvant chemotherapy (*n* = 70), Figure S2: Correlation of the overall survival with the G12D/V mutation (*n* = 70), Figure S3: Survival according to the combination of the G12D/V mutation and CA 19-9.

## Abbreviations

cfDNA: cell-free DNA, ddPCR: droplet digital polymerase chain reaction, IRB: the Institutional Review Board, PDAC: pancreatic ductal adeno-carcinoma, BMI: Body mass index, Hb: hemoglobin, WBC: white blood cell, AST: aspartate aminotransferase, ALT: alanine aminotransferase, BUN: blood urea nitrogen, CA 19-9: carbohydrate antigen 19-9, CEA: carcinoembryonic antigen, PD: pancreaticoduodenectomy, DPS: distal pancreatectomy with splenectomy, wel: well, mod: moderate, por: poor, LVI: lymphovascular invasion, PNI: perineural invasion), TNM staging: tumor, node, and metastasis staging, FAM: 6-fluorescein amidite, HEX: hexachlorofluorescein, SPSS: Statistical Package for the Social Sciences.
