# Supplementary material for: Analysis of KRAS Mutation Subtype in Tissue DNA and Cell-Free DNA Using Droplet Digital PCR and the Function of Cell-Free DNA as a Recurrence Predictive Marker in Pancreatic Cancer
Source: Biomedicines. 2021 Nov 2;9(11):1599. doi: 10.3390/biomedicines9111599 (PMC8615414; doi:10.3390/biomedicines9111599)

Supplementary material

Table S1. The fractional abundance of *KRAS* G12D and G12V mutations in the tumour tissues and blood (n = 70).

No.	Fractional Abundance (%)			
	G12D		G12V	
	Tissue	Blood	Tissue	Blood
1				
2	0.77			
3	2.7			
4	0.03		5.7	
5	3.6		0.17	
6	0.15		31.8	
7			7.6	
8			30.1	
9	0.08		0.18	
10	0.24	7.1	0.39	
11	0.07		36.6	
12	0.3		3.6	
13	15.5		0.91	
14	7.2		1.9	
15	0.11		0.32	
16	41.4		0.2	
17	0.4		9.6	1.4
18	0.04			
19	15.2		0.21	
20	3.8		0.07	
21		2.5	2.5	7
22	0.03			
23				
24	16.3	0.9	0.41	
25	0.2		5.5	
26	0.11		90.34	
27	0.51		4.8	2.6
28	0.36	0.4	28.1	
29	0.03		0.27	
30	6.3		2.8	
31			32.7	
32				
33	0.04			
34	0.11			
35			18.4	
36	0.05		42.2	3
37			0.14	
38	0.46		1.34	
39	0.06		42.1	
40	10.2		0.07	
41	0.06		0.28	
42			6.6	
43	40.9		0.09	
44	0.07		17.5	
45	0.04		5.4	
46	0.18			
47	22.7		0.11	
48	0.11		0.028	
49	0.07		30.9	
50	0.04		27.5	
51	41.5			
52	4.5		0.09	
53	0.11		0.2	2.1
54			34.4	
55	0.13		0.11	4
56	0.32		0.04	
57	17.4	4		
58	0.27		0.67	
59	1.4		34.9	2.6
60	0.08		23.4	
61	35.6		0.44	
62	39.1	3.4	0.09	
63	47.9	2.8		
64	0.09	0.7	0.21	0.6
65	51.5		0.21	
66	10.3	2.5	0.56	
67	21.4	7		
68	27.7	2.2	0.95	6
69	2.9	8	16.1	3
70	68.8	7	0.6	

Figure S1: Correlation of KRAS burden with tumour location, tumour progression, and the presence of neoadjuvant chemotherapy (n = 70). A, Fractional abundance to the tumour location. B, Fractional abundance according to the T stage. C, Fractional abundance according to the N stage. D, Fractional abundance according to the TNM stage. E, Fractional abundance according to the CA 19-9 level. F, Fractional abundance according to whether patients received neoadjuvant chemotherapy.

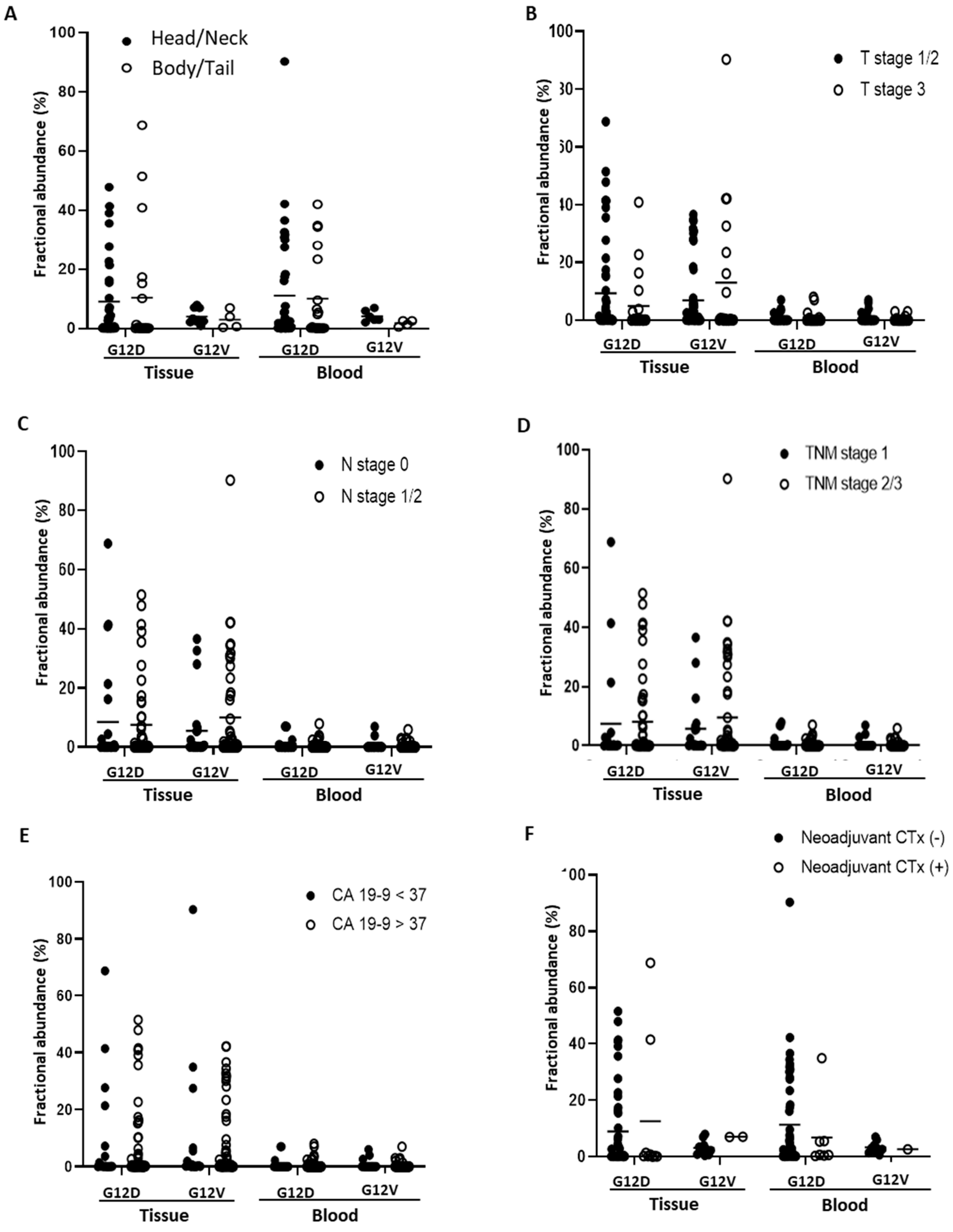

Figure S2: Correlation of the overall survival with G12D/V mutation (n = 70). A, Overall survival according to the presence of the G12D mutation in tissues. B, OS according to the presence of the G12V mutation in tissues. C, OS according to the presence of the G12D mutation in blood. D, OS according to the presence of the G12V mutation in blood (p = 0.034). E, OS according to the sum of the G12D and G12V mutations in tissues (more than 1 mutation). F, OS according to the sum of G12D and G12V mutations in tissues (more than 10, p = 0.033).

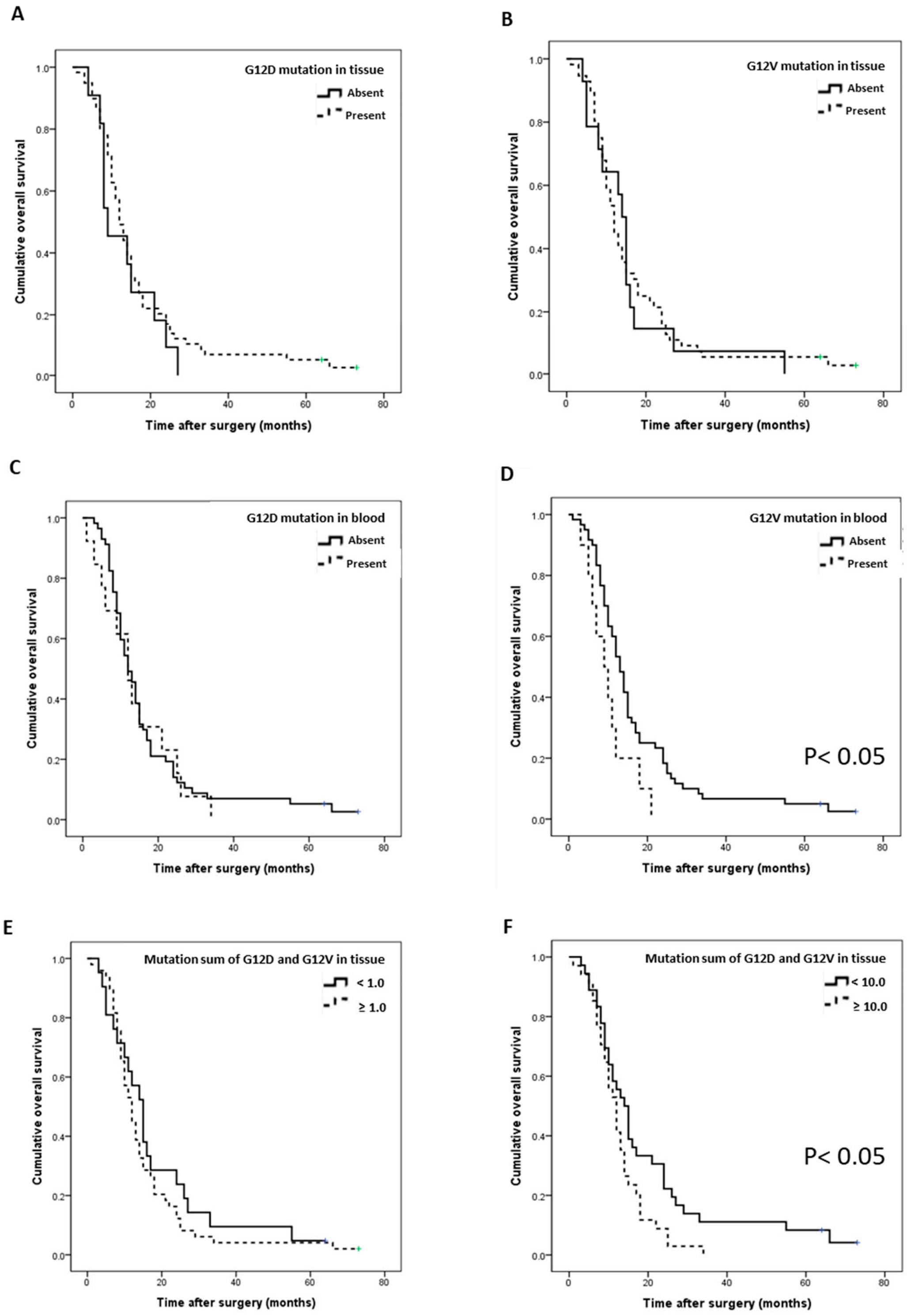

Figure S3: Survival according to the combination of G12D/V mutation and CA 19-9. A, Overall survival according to the CA 19-9 level in the blood. B, Recurrent-free survival according to the CA 19-9 level in the blood. C, OS according to the combination of G12D mutation and CA 19-9 level in tissues. D, OS according to the combination of G12V mutation and the CA 19-9 level in tissues. E, OS according to the combination of G12D mutation and the CA 19-9 level in blood. F, OS according to the combination of G12V mutation and the CA 19-9 level in blood (p = 0.014).

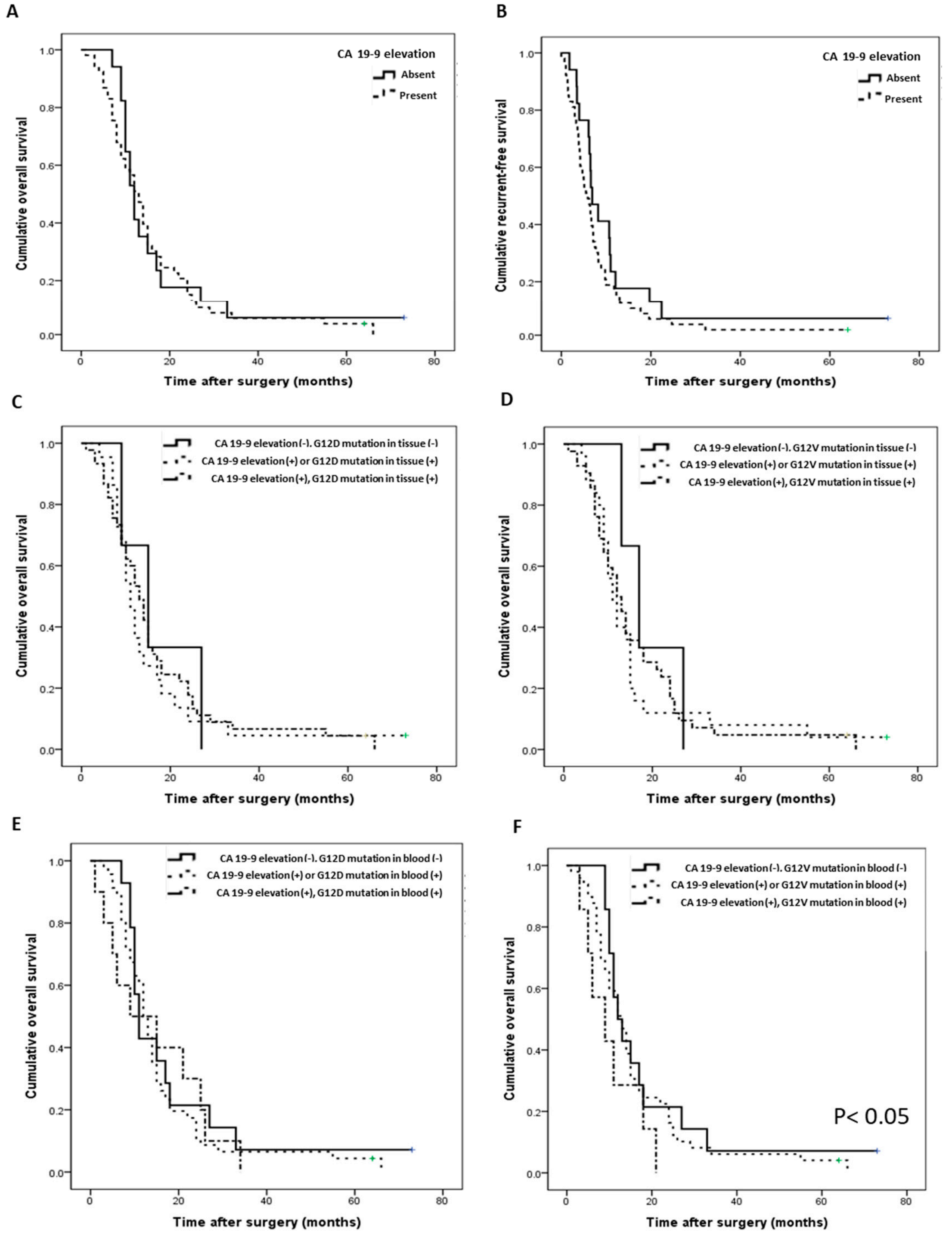

Supplement: Supplementary file 1 [file biomedicines-09-01599-s001.zip › biomedicines-1403965-supplementary.pdf]
